# Roof‐dependent atrial flutter with epicardial conduction pathway masked by left atrium posterior wall debulking ablation

**DOI:** 10.1111/anec.12997

**Published:** 2022-07-21

**Authors:** Masayuki Ishimura, Kayo Yamamoto, Masashi Yamamoto, Toshiharu Himi, Yoshio Kobayashi

**Affiliations:** ^1^ Department of Cardiology Kimitsu Central Hospital Kisarazu Japan; ^2^ Department of Cardiology Chiba University Hospital Chiba Japan

**Keywords:** atrial fibrillation, ethanol infusion, posterior wall isolation, roof‐dependent atrial flutter, septo‐pulmonary bundle, vein of Marshall

## Abstract

Roof‐dependent atrial flutter (AFL) is a major tachyarrhythmia rotating in the left atrium (LA). Here, we describe a case of roof‐dependent AFL during atrial fibrillation ablation. LA posterior wall (LAPW) debulking ablation was performed before the induction. Atrial tachycardia (AT) was induced by burst pacing, and the 3D mappings showed a focal pattern from the LA inferior area. The post‐pacing interval from the roof and bottom line corresponded to the AT cycle length. The LAPW debulking ablation masked roof‐dependent AFL due to the lack of endocardium potentials in the LAPW. We report that roof‐dependent AFL connected by epicardium fibers.

## Graphical abstract

The 3D map shows a scarred area in the left atrial posterior wall. The left atrial posterior area and mitral isthmus line act as obstructions in the tachycardia. Propagation apparently shows a centrifugal pattern from LA inferoseptal area (yellow arrows). However, the actual propagation of tachycardia is considered to detour the epicardium of the LA posterior area (yellow dotted arrow).

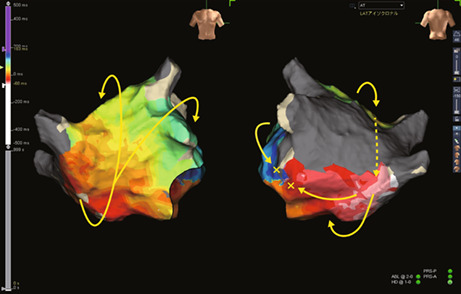

## 1 INTRODUCTION

Atrial flutter (AFL) associated with atrial fibrillation (AF) ablation is an important proarrhythmic complication (Chugh et al., 2005). Previous studies have reported several reentry patterns, including pulmonary vein (PV) gap reentry, scar‐related reentry, Marshall bundle reentry, and bi‐atrial tachycardia, associated with the existing substrate or previous ablation procedure (Hayashi et al., 2016; Kitamura et al., 2020; Ogawa et al., 2018; Satomi et al., 2008; Vlachos et al., 2019). Roof‐dependent AFL is a major reentrant tachycardia rotating in the left atrium (LA), and detailed observation using 3D mappings and electrophysiological pacing maneuvers are crucial to identify the reentry circuit of AFL (Casado et al., 2018).

Left atrium posterior wall (LAPW) isolation during AF ablation is a common procedure. The feasibility, safety, and efficacy were demonstrated in previous studies (Thiyagarajah et al., 2019). Here, we report a case where roof‐dependent AFL was masked by LAPW debulking ablation due to the lack of endocardium potentials in the LAPW. We also provide evidence that ethanol infusion into the vein of Marshall (VOM) effectively ablated epicardium fibers.

## 2 CASE REPORT

A 68‐year‐old woman with frequent episodes of palpitation was diagnosed with persistent AF. Initial catheter ablation was performed as AF was unresponsive to antiarrhythmic drug. At the time of the outpatient consultation, 12‐lead electrocardiogram revealed rapid AF with a heart rate of 160 bpm. A transthoracic echocardiogram revealed a low normal left ventricular ejection fraction of 54% and a left atrial diameter of 45 mm. The patient was administered edoxaban of 60 mg as oral anticoagulant drug and bisoprolol fumarate of 5 mg as antiarrhythmic drug. Before the ablation procedure, a single dose of edoxaban was skipped and bisoprolol fumarate was discontinued for at least five half‐lives. External cardioversion was performed by delivering 150 J in advance of catheter ablation to detect bradycardia.

The ablation procedure was performed under sinus rhythm. The strategy was comprised of PV followed by linear ablations of the roof, bottom, mitral isthmus, and cava‐tricuspid isthmus line using an irrigated tip ablation catheter (TactiCath™ contact force ablation catheter, Sensor Enabled™; Abbott) under general anesthesia. Radiofrequency pulses (30 W, 40 s, or a lesion size index of 5.0 in the PVs; 25 W, 30 s, or a lesion size index of 4.5 in the SVC; and 30 W, 40 s, or lesion size index of 5.5 in each line) were delivered point by point and supported by a steerable introducer (Agilis™ NxT steerable introducer; Abbott) to gain adequate contact force and stability. Chemical ablation using anhydrous ethanol (5 ml) infused into the VOM with posterior branch was performed before the PV isolation. Debulking ablation was used to complete the LAPW isolation because we had difficulty in LAPW isolation with roof and bottom‐line ablation. LAPW isolation was confirmed through capture loss by high‐output pacing (10 V and 1.0 ms).

**FIGURE 1:**
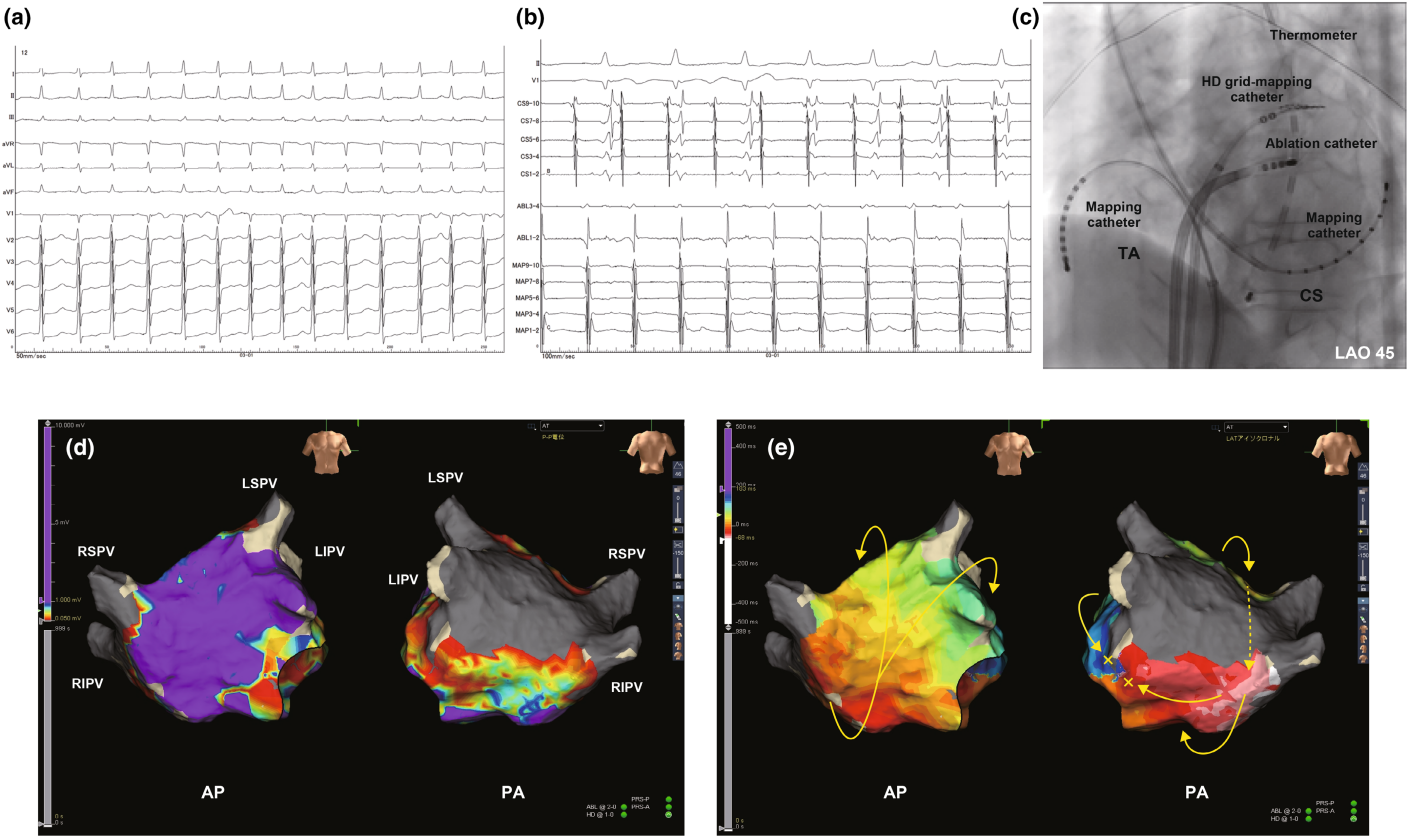
(a) 12‐lead electrocardiogram of atrial tachycardia (AT). It shows an irregular R‐R interval, where heart rate varied from 140 to 150 bpm. The polarities of P wave from each lead are flat or unclear. (b) An electrogram showing AT with a cycle length of 262 ms. In both mapping catheters with 10 electrodes, the activation sequence shows slight proximal‐to‐distal conduction. (c) Cine angiography shows the ablation catheter, the mapping catheters, including the Advisor™ HD grid mapping catheter, and thermometer. Two mapping catheters with 10 electrodes were placed in the coronary sinus and around the tricuspid annulus. LAO, left anterior oblique. (d) The bipolar voltage map during the AT. The low‐voltage and scarred areas are defined as 0.05–1.0 mV and <0.05 mV, respectively. The scarred area is observed in the pulmonary veins and left atrial (LA) posterior area, and the low‐voltage areas are identified in the LA bottom, and mitral isthmus areas. Pulmonary veins and LA posterior wall are completely isolated. AP, anteroposterior; LIPV, left inferior pulmonary vein; LSPV, left superior pulmonary vein; PA, posteroanterior; RIPV, right inferior pulmonary vein; and RSPV, right superior pulmonary vein. (e) The activation map apparently indicates centrifugal pattern from LA infero‐septal area (yellow arrows). The LA posterior area appears to act as obstructions in the tachycardia as well as the mitral isthmus line. The actual propagation of the tachycardia is considered to detour the epicardium of LA posterior area (yellow dotted arrow).

**FIGURE 2:**
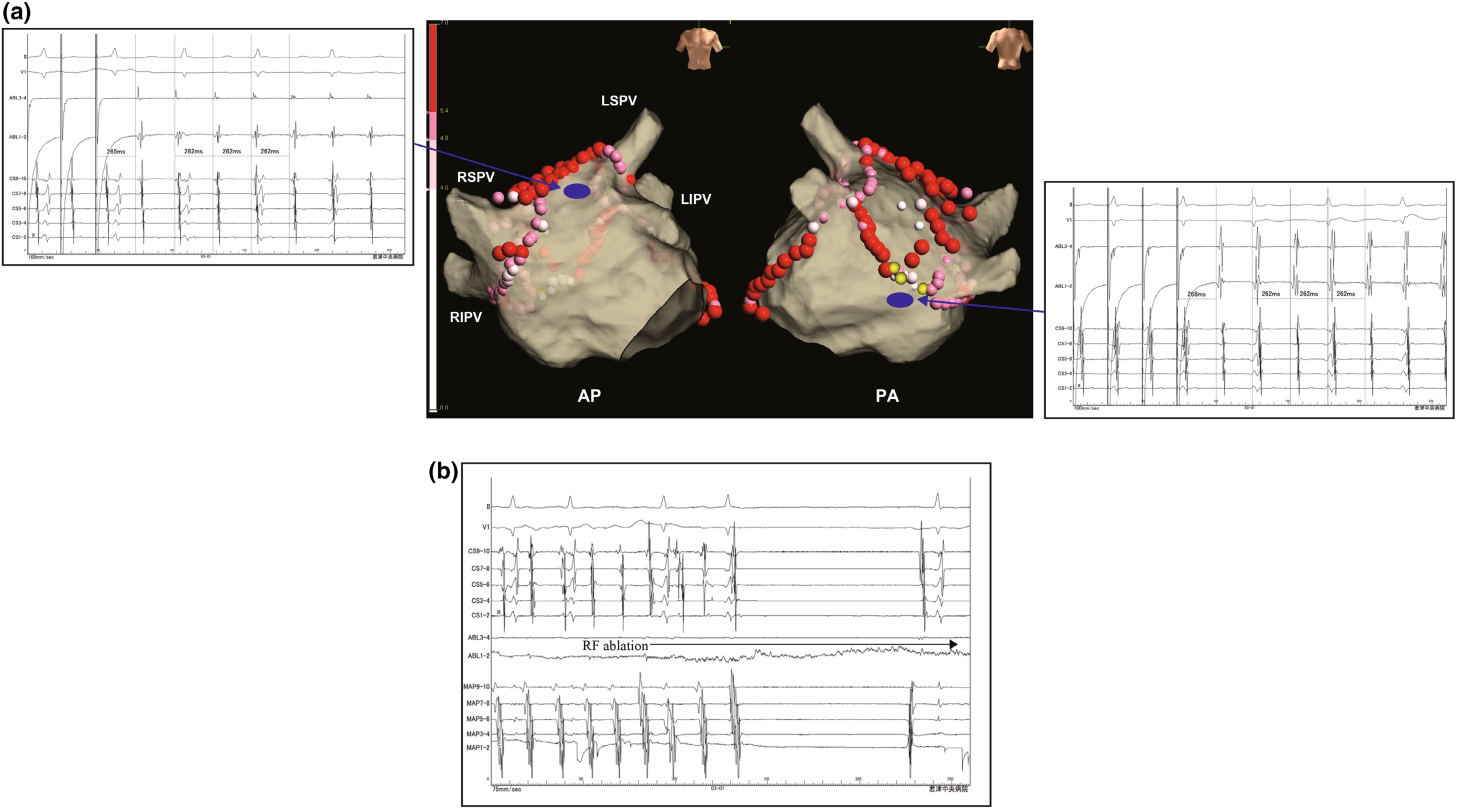
(a) Post‐pacing interval measured at the roof and bottom (blue points) at 265 and 268 ms, which closely matched tachycardia cycle length (262 ms). The tags represent ablation sites. The red, pink, and white tags show the lesion size index above 5.5, 4.0–5.5, and below 4.0, respectively. The yellow tags represent extra ablation for roof‐dependent atrial flutter. AP, anteroposterior; LIPV, left inferior pulmonary vein; LSPV, left superior pulmonary vein; PA, posteroanterior; RIPV, right inferior pulmonary vein; and RSPV, right superior pulmonary vein. (b) The second extra radiofrequency ablation (yellow tag) terminated roof‐dependent atrial flutter. RF, radiofrequency.

At the end of the procedure, atrial tachycardia (AT) was induced by atrial burst pacing (pacing cycle length of 200 ms; Figure 1a,b). The two mapping catheters placed in the coronary sinus and around the tricuspid annulus indicated the proximal‐to‐distal sequence and the tachycardia cycle length of 262 ms (Figure 1b,c). An Advisor™ HD grid mapping catheter (Abbott) and the EnSite Precision™ 2.2 software (Abbott) were used to create voltage and activation maps during the AT. The voltage map revealed scarred area in the LAPW (Figure 1d), while the activation map indicated a focal pattern wave front centrifugally propagated from the LA inferior or septal area to both atria (Figure 1e and Video S1). The LA septal area, where continuous electrical activity was observed by the mapping and ablation catheters, was initially ablated without successfully terminating AT. Subsequently, entrainment pacing was conducted to identify the reentry circuit in both atria. Post‐pacing interval (PPI) was almost matched to the AT cycle length at the LA roof, bottom line that was near to the septal area (Figure 2a). PPIs measured at the left atrial appendage, coronary sinus ostium, and crista terminalis were 388, 304, and 281 ms, respectively. Ultimately, the tachycardia was determined to be roof‐dependent AFL. We considered that the wave front detouring the epicardium of the LAPW conducted from the roof to the bottom of the LA. Additional radiofrequency applications were delivered at the bottom line (Figure 2a, yellow tags). AT was terminated during the second application and became noninducible after final ablations (Figure 2b).

## 3 DISCUSSION

Garcia et al. (2019) reported roof‐dependent AFL cases that they failed to terminate despite LAPW isolation. An epicardial component of the septo‐pulmonary bundle was also considered as an AFL conduction pathway. Miyazawa et al. (2019) also demonstrated that AFL detoured the epicardium at the anterior wall of the LA. These findings suggested that epicardial fibers could be a common conduction pathway of AFL rotating the LA. In this case, debulking ablation eliminated endocardial potentials of LAPW, and high‐output pacing (10 V and 1.0 ms) could not capture the epicardial myocardium. Therefore, the scared area masked AFL propagation in the activation map. PPIs measured at the roof and bottom line indicated the possibility that tachycardia was roof‐dependent AFL, which was confirmed by termination following ablation.

**FIGURE 3:**
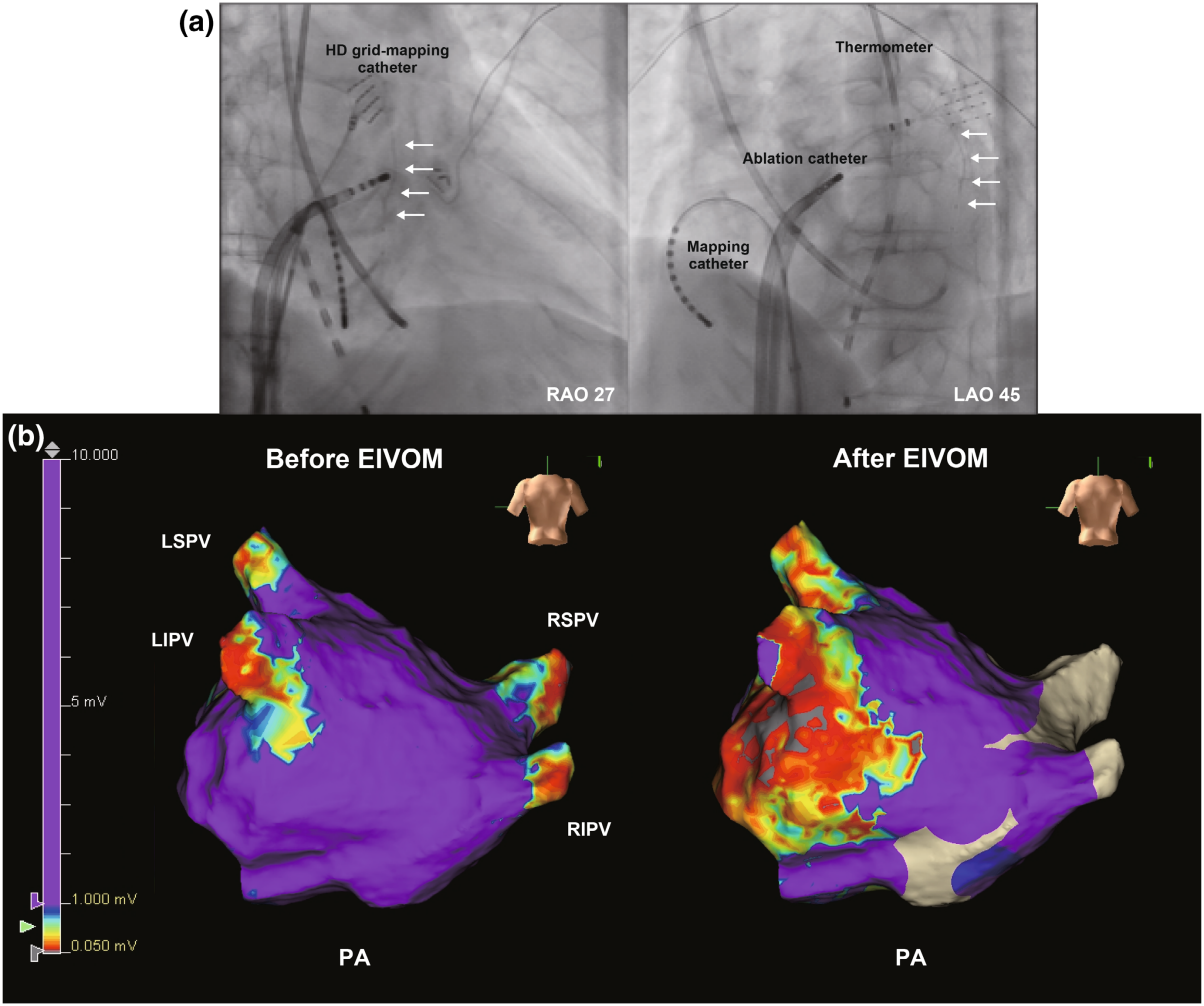
(a) Cine angiography shows a venography of the vein of the Marshall (VOM). The VOM branches (white arrows) from the great cardiac vein to posterolateral site of the mitral isthmus. LAO, left anterior oblique; RAO, right anterior oblique. (b) A 3D maps showing the left atrium voltage before and after ethanol infusion in the vein of Marshall (EIVOM). EIVOM was performed before pulmonary vein isolation. Anhydrous ethanol (5 ml) was infused into the vein of Marshall. The post‐EIVOM voltage map was obtained 1 h after EIVOM. The map shows that the posterior bottom area was ablated by EIVOM and the mitral isthmus area. AP, anteroposterior; EIVOM, ethanol infusion into the vein of Marshall; LIPV, left inferior pulmonary vein; LSPV, left superior pulmonary vein; PA, posteroanterior; RIPV, right inferior pulmonary vein; RSPV, right superior pulmonary vein.

We found that ethanol infusion into the VOM effectively ablated epicardium fibers. Jiang et al. (2019) demonstrated that epicardial potentials remained in after LAPW isolation in 33% (3/9 patients) despite endocardial capture loss. They suggested that endocardial ablation can be insufficient to make complete transmural lesion, but extra ablations along the original lines using a higher power for a longer duration could terminate AFLs. In this case, we were able to terminate AFL with two additional radiofrequency applications for the right‐side bottom line. We also found that ethanol infusion into the VOM can effectively ablate the epicardium, especially in the left‐side LAPW (Figure 3a,b). Thus, ethanol infusion might help terminate roof‐dependent AFL detouring epicardial fiber.

## 4 CONCLUSIONS

Left atrium posterior wall debulking ablation masked roof‐dependent AFL because of the lack of endocardium potentials. Ethanol infusion into the VOM is possible to effectively ablate epicardium fibers.

## AUTHOR CONTRIBUTION

Masayuki ishimura and Kayo Yamamoto conceived the idea of the case report. Masayuki Ishimura also drafted the original manuscript. Masashi Yamamoto, Toshiharu Himi, and Yoshio Kobayashi supervised the process of writing this case report. All authors reviewed the manuscript draft and revised it critically on intellectual content. All authors approved the final version of the manuscript to be published.

## CONFLICT OF INTEREST

Yoshio Kobayashi has received remuneration for lectures from Amgen Astellas BioPharma Co., Ltd., Bristol‐Myers Squibb Co., and Boehringer Ingelheim and scholarships from Medtronic Japan Co. Ltd, Daiichi Sankyo, Inc., Abbott Vascular Japan Co., Ltd., Boston Scientific Corp., Otsuka Pharmaceutical Co., Ltd., Pfizer Inc., Astellas Pharma Inc., Takeda Pharmaceutical Co., Ltd., and Japan Lifeline Co., Ltd. The rest of the authors have no conflicts of interest.

## ETHICAL APPROVAL

This case report was approved by the Institutional Review Board of Kimitsu Central Hospital and was conducted according to the principles of the Declaration of Helsinki.

## Supporting information


Video S1

## Data Availability

The data that support the findings of this study are available on request from the corresponding author. The data are not publicly available due to privacy or ethical restrictions.
